# Fractioning of Proanthocyanidins of *Uncaria tomentosa*. Composition and Structure-Bioactivity Relationship

**DOI:** 10.3390/antiox6030060

**Published:** 2017-07-28

**Authors:** Mirtha Navarro, William Zamora, Silvia Quesada, Gabriela Azofeifa, Diego Alvarado, Maria Monagas

**Affiliations:** 1 Department of Chemistry, University of Costa Rica (UCR), Sede Rodrigo Facio, San Pedro de Montes de Oca, San José 2060, Costa Rica; mnavarro@codeti.org (M.N.); wzamorra10@alumnes.ub.edu (W.Z.); 2 Department of Biochemistry, Faculty of Medicine, University of Costa Rica (UCR), Sede Rodrigo Facio, San Pedro de Montes de Oca, San Jose 2060, Costa Rica; silvia.quesada@ucr.ac.cr (S.Q.); gabriela.azofeifacordero@ucr.ac.cr (G.A.); 3 Department of Biology, University of Costa Rica (UCR), Sede Rodrigo Facio, San Pedro de Montes de Oca, San Jose 2060, Costa Rica; luis.alvaradocorella@ucr.ac.cr; 4 Institute of Food Science Research (CIAL), Spanish National Research Council (CSIC-UAM), C/Nicolás Cabrera 9, Madrid 28049, Spain

**Keywords:** *U. tomentosa*, UPLC, TQ-ESI/MS, ^13^C-NMR, proanthocyanidins, propelargonidins, procyanidins, mass spectrometry, antioxidant, cytotoxicity

## Abstract

In a previous study, the detailed low-molecular weight polyphenolic profile of the different plant parts (leaves, stem, bark and wood) of *Uncaria tomentosa* was reported, the leaves being the plant part with the highest phenolic content and presenting the most heterogenous proanthocyanidin composition. Further, cytotoxicity of leaves extracts in two cancer cell lines was also found to be higher than in the remaining parts of the plant. In the present study, fractioning of *U. tomentosa* leaves polyphenolic extracts was performed using Diaion^®^ HP-20 resin and a detailed characterization and quantification of fractions (*n* = 5) was achieved using advanced analytical techniques such as Ultra-Performance Liquid Chromatography coupled with Electrospray Ionization and Triple Quadrupole (TQD) Tandem Mass Spectrometry (UPLC/TQ-ESI-MS) and ^13^C-NMR. Oxygen Radical Absorbance Capacity (ORAC) and cytotoxicity on gastric adenocarcinoma AGS and colon adenocarcinoma SW20 cell lines were also determined in the different fractions. Results showed selective distribution of 32 non-flavonoid and flavonoid phenolics among the different fractions. ORAC varied between 3.2 and 11.8 μmol TE/mg in the different fractions, whereas IC_50_ of cytotoxicity on gastric adenocarcinoma AGS and colon adenocarcinoma SW20 cell lines best values were between 71.4 and 75.6 µg/mL. Fractions rich in proanthocyanidins also showed the highest bioactivity. In fact, significant positive correlation was found between total proanthocyanidins (TP) quantified by UPLC-DAD and ORAC (*R*^2^ = 0.970), whereas significant negative correlation was found between TP and cytotoxicity towards AGS (*R*^2^ = 0.820) and SW620 (*R*^2^ = 0.843) adenocarcinoma cell lines. Among proanthocyanidins, propelargonidin dimers were of particular interest, showing significant correlation with cytotoxic selectivity on both gastric AGS (*R*^2^ = 0.848) and colon SW620 (*R*^2^ = 0.883) adenocarcinoma cell lines. These results show further evidence of the bioactivity of *U. tomentosa* proanthocyanidin extracts and their potential health effects.

## 1. Introduction

Proanthocyanidins, constituted by condensed flavan-3-ols units, have been the object of different studies due to their potential health benefits, mainly considering their antioxidant activity and the important role these may play by acting as cancer chemopreventive, anti-inflammatory agents, and by reducing risk of cardiovascular mortality [1]. However, the characterization as well as the determination of the content and distribution of proanthocyanidins in botanicals, and derived dietary supplements and herbal medicines is a very difficult task [2].

Despite the increasing number of studies on polyphenols and their anticancer effects, there is a general recognition of the need for further knowledge on the mechanisms and factors that influence these effects, such as the presence of direct antioxidant activity, the promotion of endogenous defenses against oxidative stress, epigenetic regulation by modulating microRNA (miRNA) and direct protein interaction [3]. Some studies report a correlation between polyphenolic contents and cytotoxicity and selectivity towards cancer cell lines [4,5], while other studies claim that there is no correlation or selectivity [6,7].

A wide array of bioactivities has been reported for *Uncaria tomentosa* (Cat’s claw) extracts, including immunostimulant, anti-inflammatory, antioxidant properties, and protective effects against cancer [8,9], with studies attributing these properties to different constituents such as alkaloids and terpenes [10]. Other studies suggest that phenolic compounds could be partially responsible for some of these effects, for instance, its antioxidant properties [11,12].

Recently, the low-molecular weight polyphenol profile of extracts from different parts of *U. tomentosa* was published by our group showing that leaves are composed of heterogenous proanthocyanidins, also showing the highest phenolic content among the different plant parts [13]. Moreover, other studies also indicated the proanthocyanidin contents of leaves extracts to be an important factor on different bioactivities, including antioxidant capacity and cytotoxicity on gastric and colon adenocarcinoma cell lines [14]. Bioactivity of proanthocyanidins on epithelial gastrointestinal cancer cells lines could be highly relevant since, because of their low absorption, gut epithelial cells are likely one of the main tissues where these compounds can actually exert their biological effects [15]. Therefore, considering all these facts, the objective of the present work was to obtain enriched polyphenolic fractions of *U. tomentosa* leaves exhibiting different polyphenol distribution and content, and to evaluate their in vitro bioactivity in order to establish a possible structure-bioactivity relationship. For this purpose, we have characterized five different fractions by UPLC-DAD-ESI-TQ MS and ^13^C-NMR techniques, and determined their Oxygen Radical Absorbance Capacity (ORAC) and the antitumoral effects in gastric and colon cancer cell lines. 

## 2. Materials and Methods

### 2.1. Plant Material, Chemicals and Reagents

*Uncaria tomentosa* leaves were collected from Los Chiles, Costa Rica, and the voucher (series no. AQ3332) is deposited in the Costa Rican National Herbarium. After drying the leaves in a stove at 40 °C, the material was ground and preserved at −5 °C. Chloroform, methanol and MTBE (methyl tert-butyl ether) solvents were obtained from Baker (Center Valley, PA, USA) and DMSO (dimethyl sulfoxide) from Sigma-Aldrich (St. Louis, MO, USA). Diaion^®^ HP-20 resin, Trypsin-EDTA solution, 2,2′-azobis(2-methyl-propionamidine)-dihydrochloride (AAPH) and 3-(4,5-dimethylthiazol-2-yl)-2,5-diphenyltetrazolium bromide (MTT) reagents were also purchased from Sigma-Aldrich (St. Louis, MO, USA). Finally, glutamine, MEM (Minimum essential Eagle’s medium with 10% fetal bovine serum (FBS)), penicillin-streptomycin antibiotics and amphotericin B antifungal were provided by Life Technologies (Carlsbad, CA, USA). 

### 2.2. Extraction and Fractioning of Phenolic Compounds 

The phenolic extract from *U. tomentosa* leaves (10.4 g extract/100 g dried material) was obtained as previously described [13]. Briefly, non-polar compounds were extracted in a mixture (0.05 mg/mL) of MTBE and methanol (MeOH) 90:10 (*v*/*v*) at 25 °C for 24 h, and, after filtration, the extraction process was repeated once. Afterwards, the polyphenolic-rich extract was obtained by extracting the residual plant material with MeOH at 25 °C for 24 h, and after filtration, the extraction process was repeated twice. The methanol extracts were combined and evaporated to dryness, under 40 °C. The polyphenolic extract was fractionated using a Diaion^®^ HP-20 resin. Briefly, 8 g of the *U. tomentosa* leaves extract were dissolved (0.05 g/mL) in a mixture of methyl tert-butyl ether (MTBE) and methanol (MeOH) 70:30 (*v*/*v*) at 25 °C; and separated in a column where 72 g of resin were packed with methanol and then washed with distilled water to remove methanol completely. The extract was loaded in the column and eluted with 500 mL each of a mixture of water and MeOH 90:10 (*v*/*v*), 70:30 (*v*/*v*), 50:50 (*v*/*v*), 30:70 (*v*/*v*), 10:90 (*v*/*v*), finalizing with 500 mL of 100% methanol. Fractions were evaporated to dryness in a rotavapor.

### 2.3. Analysis of Phenolic Compounds by UPLC-DAD-ESI-TQ MS

The UPLC-HRMS system used to analyze the samples consisted of an Acquity TQD tandem quadrupole mass spectrometer with Z-spray electrospray interface coupled with an Acquity PDA eλ photodiode array detector (DAD) (Waters^®^, Milford, MA, USA). Separation was carried out using a BEH C18 UHPLC column (2.1 × 100 mm; 1.7 µm, Waters^®^) at a flow rate of 0.5 mL/min and at 40 °C. Sample injection volume was 2 µL (5 mg/mL in CH_3_CN:H_2_O 1:4). Mobile phases A and B consisted of a combination of water:acetic acid (98:2, *v*/*v*) and acetonitrile:acetic acid (98:2, *v*/*v*), respectively. The gradient started with 0.1% B (*v*/*v*) held to 1.5 min, then varied from 0.1% to 16.3% B at 11.17 min, to 18.4% B at 11.5 min, and held at 18.4% B to 14 min. After, it went to 99.9% B at 14.1 min, held at 99.9% B to 15.5 min, returning to 0.1% B at 15.6 min and held at 0.1% B to 18 min. Negative electrospray ionization mode was used under conditions set as follows: desolvation gas (N_2_) flow rate, 750 L/h; desolvation temperature, 400 °C; cone gas (N_2_) flow rate, 60 L/h, capillary voltage, 3 kV; source temperature, 130 °C. Multiple reaction monitoring (MRM) mode was used for quantification [16,17], using external calibration curves for the following transitions: (+)-catechin and (−)-epicatechin (*m*/*z* 289/245), procyanidin dimers (*m*/*z* 577/289), procyanidin trimers *m*/*z* 865/577), propelargonidin dimers (*m*/*z* 561/289), and cinchonains (*m*/*z* 451/289). (+)-Catechin, (−)-epicatechin, procyanidin B1, B2, and C1 commercial standards were used (Sigma-Aldrich), while procyanidin B3, B5, B7, and T2 standards were isolated from plants and assigned by MS/MS analysis. Propelargonidins were quantified with (−)-epicatechin calibration curve and cinchonains with the one from procyanidin B1. The UV/Vis spectra were recorded between 250–420 nm.

### 2.4. Analysis by ^13^C-NMR

^13^C-Nuclear Magnetic Resonance (NMR) data were acquired at 100 MHz on a Bruker Ascend 400 spectrometer, using CD_3_OD as solvent.

### 2.5. In Vitro Antioxidant Activity

The ORAC method [18] was used to measure the fluorescence of a mixture of AAPH (12 mM), fluorescein (70 nM), and different concentrations of antioxidant (Trolox standard or phenolic sample dissolved in CH_3_OH (0.1% HCl *v*/*v*)), in phosphate buffer (75 mM, pH 7.4) at 37 °C, using black 96-well untreated microplates (Nunc, Denmark). A Polarstar Galaxy plate reader (BMG Labtechnologies GmbH, Offenburg, Germany) with a Fluostar Galaxy software (v.4.11-0, BMG Labtechnologies GmbH, Offenburg, Germany) was used to record fluorescence every minute during 98 min. Three independent runs were performed for each sample. Fluorescence values were normalized (to the blank (no antioxidant) curve) in order to obtain the area under the fluorescence decay curve (AUC). Finally, the regression equation between antioxidant concentration and net AUC (calculated in respect to the blank) was calculated to estimate the ORAC value (slope of sample equation divided by slope of Trolox equation), which is expressed as mmol of Trolox equivalents (TE)/g of phenolic sample.

### 2.6. Assessment of Cytotoxicity

The human colorectal adenocarcinoma cell line SW 620, human gastric adenocarcinoma AGS, and monkey normal epithelial kidney cells Vero (American Type Culture Collection (ATCC), Rockville, MD, USA), were grown in MEM (10% FBS) containing 100 IU/mL penicillin, 100 µg/L-streptomycin, 0.25 µg/mL amphotericin B and 2 mmol/L glutamine (in an atmosphere with 5% CO_2_ at 37 °C). To perform the cytotoxicity assays, 100 µL of cell suspension (1.5 × 10^5^ cells/mL) were seeded overnight into 96-well plates to obtain 100% confluent cells in each well. Then, the cells were exposed for 48 h to 50 µL of *U. tomentosa* fractions (15–500 µg/mL final concentration in MEM (DMSO 0.1% *v*/*v*)). Afterwards, MEM was removed, cells washed with Phosphate-Buffered Saline (PBS), and 100 µL of a MTT solution in PBS (0.5 mg/mL final concentration) were added and incubated for 2 h at 37 °C, to obtain insoluble formazan. Then, MTT was eliminated and absorbance of a solution of formazan crystals dissolved in 100 µL of ethanol 95% was measured at 570 nm. To prepare the control wells used to determine the 100% of viability; DMSO was diluted in media in the same way as the fractions and incubated with the cells for 48 h. IC_50_ (minimum concentration required for 50% inhibition of cells viability) were obtained from dose-response curves for each sample. Different doses of extracts were analyzed in triplicate and three independent experiments were run for each extract and cell line. Selectivity index (SI) was determined in order to evaluate if the cytotoxicity was specific against the cancer cells. This index is defined as the ratio of IC_50_ values of normal epithelial kidney cells (Vero) to cancer cells (SW620 or AGS). Samples with SI greater than 3 are considered to have a high selectivity towards cancer cells [19].

## 3. Results

### 3.1. Polyphenolic Composition by UPLC/TQ-ESI-MS

UPLC/TQ-ESI-MS analysis was performed in the seven fractions obtained from *U. tomentosa* leaves extract, as described in the experimental section. Preliminary analysis indicated that only the last five fractions, namely LH-F3 to LH-F7, were rich in polyphenols. A total of 32 polyphenols were identified, grouped as non-flavonoid phenolics (hydroxybenzoic acids and hydroxycinnamic acids); flavan-3-ols ((epi)catechin monomers, procyanidin dimers, propelargonidin dimers, procyanidin trimers and flavalignans (cinchonains)). Among procyanidins, B1, B2, B3, B4, B5, B7 dimers and C1, T2 trimers were found as well as four propelargonidin dimers (with retention times of 4.43, 5.01, 5.65 and 9.27 min). Table 1 summarizes the polyphenolic profile and content (in µg/g extract) of the polyphenolic-rich fractions LH-F3 to LH-F7. Table S1 (Supplementary Materials) reports MS/MS parameters and Figure S1 (Supplementary Materials) shows MRM transitions for proanthocyanidins, including procyanidin dimers (*m*/*z* 577/289), propelargonidin dimers (*m*/*z* 561/289), procyanidin trimers (*m*/*z* 865/577) and flavalignans-cinchonains (*m*/*z* 451/341).

Hydroxycinnamic acids were the less abundant compounds in the different fractions followed by hydroxybenzoic acids. Fractions LH-F6 and LH-F7 presented the largest proportion of these compounds (3.5–3.9%, and 7.4–8.4%, of hydroxycinnamic and hydroxybenzoic acids respectively), whereas as LH-F3 was devoid from hydroxycinnamic acids and just 0.6% of hydroxybenzoic acids. In relation to flavan-3-ols, both monomers were present in all samples, (−)-epicatechin being found in larger concentration than (+)-catechin. Procyanidin dimers and propelargonidin dimers were the most abundant flavan-3-ols, representing from 11.4% o in LH-F7 to 77.5% in fraction LH-F3. In contrast, the content of flavalignans presented the opposite trend, representing 28.5% of total polyphenols in LH-F4, and increasing up 75.5% in LH-F7. Among proanthocyanidins (PA), procyanidin dimer B4 [(+)-(catechin-(4α→8)-(−)-epicatechin] was the most abundant procyanidin in the different fractions, especially in LH-F3 (20.6% of PA) followed by B3 [(+)-catechin-(4α→8)-(+)-catechin], which also showed the highest proportion in fraction LH-F3 (10.6% of PA). Propelargonidin dimer at 4.47 min was the most abundant, especially in fraction LH-F3 (20.0% of PA), followed by propelargonidin dimer at 5.74 min, in particular in fraction LH-F4 (17.6% of PA).

### 3.2. ^13^C-NMR Analysis of U. tomentosa Fractions

**Figure 1:**
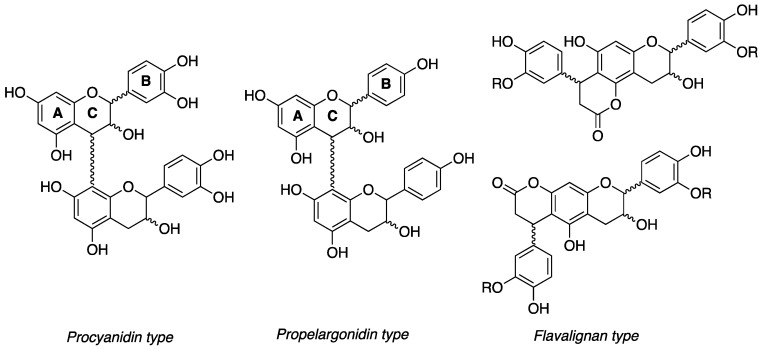
Procyanidin (PC), propelargonidin (PP), and flavalignans (FL) general chemical structures.

We have previously reported the analysis of ^13^C-NMR spectra for *U. tomentosa* extracts, to complement the characterization of polyphenols by UPLC-MS/MS [13]. Here, in a similar way, we have carried out ^13^C-NMR spectra measurements of the fractions obtained from *U. tomentosa* leaves. Fractions LH-F3 to LH-F7 showed signals characteristic of proanthocyanidins (Figure 1), as previously reported in other substrates containing procyanidins [20] and propelargonidins [21]. These results are also in agreement with UPLC results found in the different fractions.

**Figure 2:**
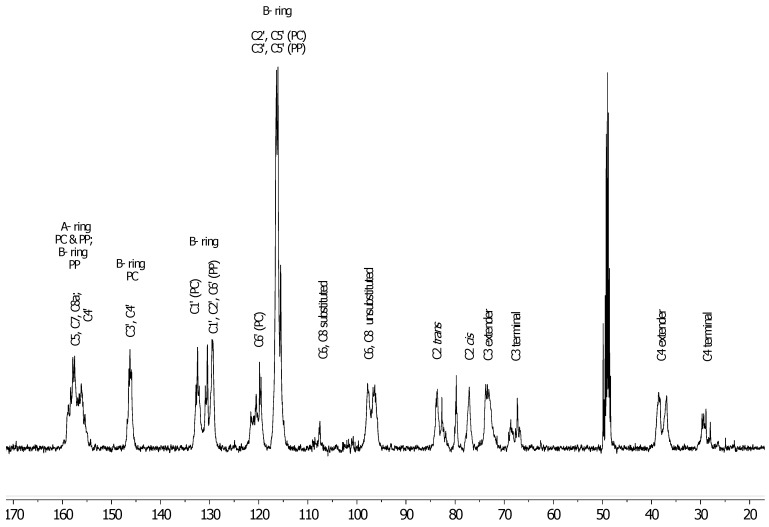
^13^C-NMR (MeOD) for a polyphenolic fraction of leaves (LH-F3) from *U. tomentosa.*

As an example, Figure 2 illustrates the spectra obtained for LH-F3. Signals observed between δ 160 and 150 ppm can be attributed to carbon C4’ of the B ring for propelargonidins (PP) and to carbons C5, C7, C8 of the A ring from both procyanidins (PC) and propelargonidins (PP) [20,21].

The chemical shifts for characterizing B ring resonances of polyphenols appear in the range of δ 150–115 ppm, some of which are useful for distinguishing a specific type of this kind of compound. For instance, PC presented characteristic signals at δ 146.2 ppm (C3’ & C4’ of the B ring), δ 132.5 ppm (C1’ of the B ring) and δ 119.8 ppm (C6’ of the B ring). On the other hand, PP gave distinctive signals at δ 130.4 ppm (C1’ of the B ring) and at δ 129.4 ppm (C2’ & C6’ of the B ring). The cluster of peaks between δ 117–115 ppm were attributed to C2’, C5’ (PC) and C3’, C5’ (PP). 

The upfield shifts from B ring resonances (signals to the right 115 ppm) provided two main pieces of structural information. First, the stereochemistry of C ring, where the signals to δ 83.7 ppm and 77.1 ppm for C2 were assigned of both isomers, 2,3-*trans* (catechin and afzelechin units) and 2,3-*cis* (epicatechin and epiafzelechin units), respectively. Second, the confirmation of the formation of polymeric structures due to the broad peaks at δ 107.6 ppm (bonded monomer units C4µ→C8 and C4→C6), δ 73.4 ppm (C3 extended units) and δ 39–36 ppm (C4 extended units). 

The ^13^C-NMR spectra for the other fractions (LH-F4 to LH-F7) also presented structural information corresponding to the specific phenol content of these fractions (Figure S2, Supplementary Materials). For instance, the ^13^C-NMR spectra for Fraction LH-F4 was very similar to the one described above, due to the high content of PP (39.2%) and PC (21.9%), that can be noted in the signals between δ 160 and 150 ppm. Fraction LH-F5 presented mainly signals characteristic for PP (δ 130–129 ppm, B ring) whereas Fraction LH-F6 and LH-F7 showed mainly signals corresponding to flavalignan-cinchonains (FL), for instance at δ 135.5 ppm consistent with phenylpropanoid units.

### 3.3. Antioxidant Activity

Table 2 summarizes the antioxidant activity results for the *U. tomentosa* fractions along with the total proanthocyanidin (TP) (i.e., calculated as the sum of procyanidin dimers, propelargonidin dimers, procyanidin trimers and flavalignan-cinchonains) content for each fraction, obtained by UPLC/TQ-ESI-MS (Table 1). Among the different fractions, LH-F3 showed the highest antioxidant value and LH-F7 the lowest one, which is in agreement with the respective TP content. Further, a correlation analysis was carried out between the ORAC values and the total proanthocyanidin content and a significant positive correlation was observed (*r* = 0.987, *p* < 0.05). A correlation analysis between ORAC and each phenolic group’s content was also performed and a significant positive correlation was found for propelargonidin dimers (*r* = 0.895, *p* < 0.05). At the individual level, a significative positive correlation with ORAC values (*r* = 0.998, *p* < 0.05) was found for three compounds, namely propelargonidin dimer at 4.47 min, procyanidin B2 and procyanidin B4.

### 3.4. Cytotoxicity of U. tomentosa Fractions 

Cytotoxicity results are summarized in Table 3 (Figures S3 and S4 in Supplementary Materials, show dose-response curves). IC_50_ values indicated that cytotoxicity of *U. tomentosa* extracts were dependent on the cancer cell line used and was also influenced by the phenolic composition of the different fractions. Fractions LH-F3, LH-F4 and LH-F5 showed the most promising results including important cytotoxicity in both cancer cell lines (IC_50_ < 130 µg/mL) and a selectivity index (SI) showing values greater than 3.5. As mentioned, selectivity could suggest an apoptotic effect directed towards cancer cells with respect to normal cells, which is important for any chemotherapeutic use [19,22].

The lower cytotoxicity shown by fractions LH-F6 and LH-F7 coincides with lower contents in procyanidin and propelargonidin oligomers observed in these fractions. In contrast, fractions LH-F3, LH-F4 and LH-F5 showing larger concentrations of these compounds also exhibited higher cytotoxicity towards both gastric AGS and colon SW620 adenocarcinoma cell lines. Whereas growth of normal Vero cells was not affected by *U. tomentosa* extracts LH-F3 and LH-F4 (IC_50_ > 500 µg/mL), it was affected by the remaining three extracts. Particularly, fraction LH-3 exhibited remarkable cytotoxicity on both adenocarcinoma cell lines (IC_50_ = 75.6 and 71.4 µg/mL, respectively, for AGS and SW620 cells) with highest selectivity, which is in agreement with the largest content of properlagonidins and procyanidins present in this fraction.

**Figure 3:**
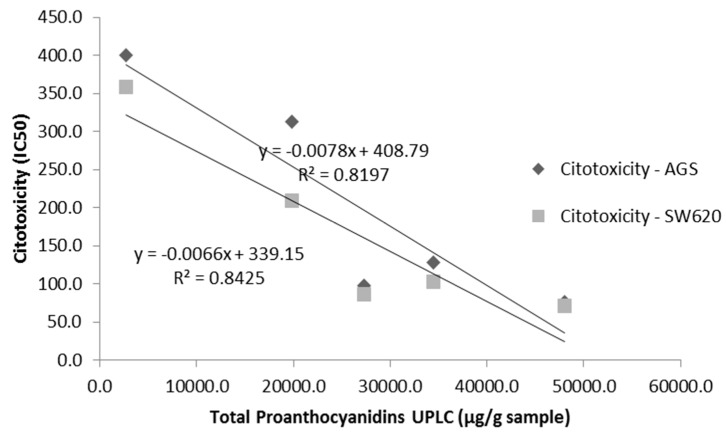
Correlation between total proanthocyanidin contents (UPLC/TQ-ESI-MS) and cytotoxicity on AGS and SW620 adenocarcinoma cell lines.

A correlation analysis was carried out between total proanthocyanidins (TP, UPLC/TQ-ESI-MS), polyphenolic groups contents (UPLC/TQ-ESI-MS), ORAC and cytotoxicity on both adenocarcinoma AGS and SW620 cell lines. A significant negative correlation was found between TP and both cancer cell lines, gastric AGS adenocarcinoma (*R*^2^ = 0.820) and colon SW620 adenocarcinoma (*R*^2^ = 0.843) (Figure 3), indicating that lower IC50 values were obtained with higher contents of proanthocyanidins, and at the same time suggesting that these compounds exert more potent cytotoxic effects. In turn, correlation between ORAC antioxidant capacity values and cytotoxicity was cell line-dependent, with a significant negative correlation observed in the case of colon SW620 adenocarcinoma cell line (*R*^2^ = 0.786), but a significant correlation was not found for AGS cells (data not shown).

**Figure 4:**
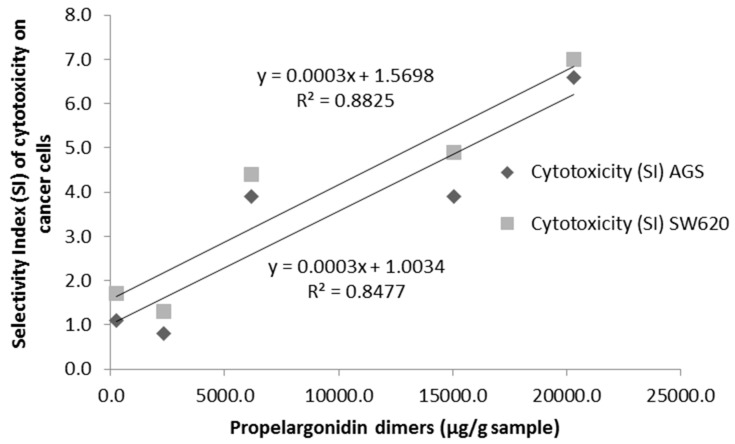
Correlation between propelargonidin dimers contents (UPLC/TQ-ESI-MS) and selectivity index (SI) of cytotoxicity on AGS and SW620 adenocarcinoma cell lines.

Finally, in relation to the selectivity of cytotoxicity towards cancer cell lines in respect to normal (vero) cells, the analysis indicated a significant positive correlation between the content of propelargonidin dimers and both cancer cell lines, gastric AGS adenocarcinoma (*R*^2^ = 0.848) and colon SW620 adenocarcinoma (*R*^2^ = 0.883) (Figure 4), suggesting that higher contents of propelargonidin dimers are associated with higher selectivity towards both adenocarcinoma cell lines. Our findings also indicated that the content of procyanidin dimers has a cell line-dependent correlation with selectivity of cytotoxicity (SI), with a significant positive correlation in the case of AGS adenocarcinoma cell line (*R*^2^ = 0.785); whereas no correlation was found for SW620 cells.

## 4. Discussion

**Figure 5:**
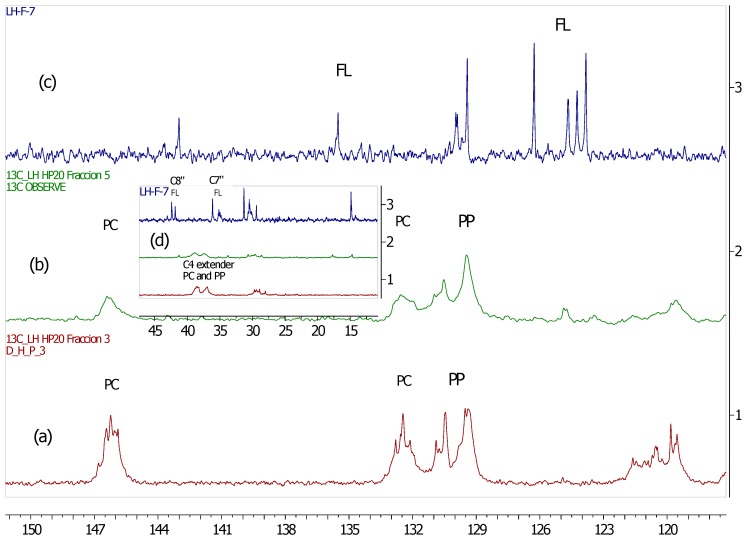
^13^C-NMR (MeOD) for different fractions of leaves from *U. tomentosa:* (**a**) fraction LH-F3; (**b**) fraction LH-F5; (**c**) fraction LH-F7 and (**d**) amplification for the three fractions in the range of δ 45–15 ppm.

^13^C-NMR spectra allowed to confirm the presence of the main type of proanthocyanidin (procyanidins, propelargonidins and flavalignans) found in this study by UPLC-MS/MS. In fact, Figure 5 shows amplification of ^13^C-NMR spectra for three different fractions (LH-F3, LH-F5 and LH-F7) of *U. tomentosa* leaves. The fraction LH-F3 contained a significant amount of procyanidins (PC) (46%) and propelargonidins (PP) (37%), and, indeed, the assignment of distinctive signals for these compounds was clearly shown in Figure 5a. On the other hand, fraction LH-F5 (Figure 5b) contained a higher amount of PP (21%) than PC (6%), and according stronger signals at δ 130–129 ppm (C2’, C6’ and C1’ of the B ring in PP) in respect to the cluster at δ 146–145 ppm (C3’ & C4’ of the B ring in PC) were observed. Finally, flavalignans (FL) were the main compounds present (76%) in fraction LH-F7 (Figure 5c), and indeed, the characteristic signals were detected at δ 135.5 ppm corresponding to C1” [23], and δ 127–123 ppm that likely correspond to chemical shifts of C6” and/or C6’ when the hydroxyl in C3” and/or C3’ have a methyl group, whereas characteristic resonances of the aromatic methoxy groups occur in the range δ 58–54 ppm. Other distinguishing signals for flavalignans were found around δ 42.4 ppm (two peaks) and δ 36.1 ppm (see Figure 5d) that can be attributed to methylene C8” and the methine C7”, respectively. In sum, the specific phenolic composition and the relative percentages between those compounds in each fraction generate characteristic ^13^C-NMR spectra, which allows corroborating the results delivered by UPLC-MS.

In respect to bioactivity, the results of the evaluation of antioxidant capacity ORAC are in agreement with studies reporting a correlation between total polyphenolic contents and antioxidant activity [24]. For instance, Dudonné et al. [25] reported a correlation (*R*^2^ = 0.736) between total phenolic content and ORAC values for different plant extracts. Our results are more specific, indicating not only significant positive correlations with total proanthocyanidin contents (*R*^2^ = 0.970), but also with particular structures such as propelargonidin dimers (*R*^2^ = 0.790). Regarding cytotoxicity, the mechanisms underlying the effect of polyphenols extracts in some cancer cells remain to be elucidated, due to factors such as modulation of signaling pathways and epigenetic regulation of gene expression that are important modulators of cancer cell phenotypes, their effect in Reactive Oxygen Species (ROS) homeostasis, inhibition of mutagenesis, upregulation of mechanisms of DNA maintenance [26,27,28], and the fact that these molecules can reach multiple targets, such as membrane receptors, signaling proteins, miRNA expression, chemokines and inflammatory mediators [3], suggesting that polyphenols could work in a synergistic manner. In agreement with our results, previous studies showed that proanthocyanidins extracts from cranberries inhibited proliferation in colon and prostate cancer cell lines [29] and extracts from grape seeds exhibited similar cytotoxic effect in cervic cancer [30]. In fact, our findings indicate that polyphenolic fractions LH-F3 and LH-F4 from *U. tomentosa* leaves, containing a richer mixture of proanthocyanidins dimers, are the ones more cytotoxic and also more selective towards both gastric AGS and colon SW620 adenocarcinoma cell lines with respect to normal vero cells. These results are in agreement with studies showing selective inhibition on the proliferation of breast cancer cell lines like MDA and MB-231 using an enriched fraction of proanthocyanidins [5] or others, demonstrating that an enriched fraction of these compounds exhibit differential cytotoxicity between colon cancer and normal gastric mucosal cell lines [31]. In contrast, fractions LH-F6 and LH-F7 did not show selectivity between cancer and normal cells, similarly to other studies reporting that proanthocyanidin extracts are equally cytotoxic to tumourigenic and normal breast and prostate cell lines [7]. Moreover, we found a significant negative correlation of total proanthocyanidins (UPLC/TQ-ESI/MS) with cytotoxicity on both gastric AGS and colon SW620 adenocarcinoma cell lines (Figure 3), suggesting a higher proanthocyanidin contents effect on lower IC50 values associated with greater cytotoxicity. Regarding selectivity of cytotoxicity towards cancer cells, a significant positive correlation was found for propelargonidin dimers contents on both cell lines (Figure 4), indicating the possible role of these compounds on higher selectivity. Finally, results showed cell-dependence for procyanidin dimers, since a positive correlation was observed for selectivity of cytotoxicity in AGS gastric cells (*R*^2^ = 0.786), while no correlation was found for SW620 colon cells. In sum, our findings are in agreement with polyphenols acting in a synergistic manner, and with the fact that potential selectivity on cells could be dependent on the structure of proanthocyanidins.

## 5. Conclusions

This paper describes a successful method to obtain enriched polyphenolic fractions of *U. tomentosa*. Using advanced analytical techniques such as UPLC/TQ-ESI-MS and ^13^C-NMR, results showed selective distribution of 32 non-flavonoid and flavonoid phenolics among the fractions, proanthocyanidin being predominant in those fractions with higher bioactivity. In fact, a significant positive correlation was found between proanthocyanidin contents and ORAC antioxidant capacity (*R*^2^ = 0.970), as well as significant negative correlations with cytotoxicity on AGS (*R*^2^ = 0.820) and SW620 (*R*^2^ = 0.843) adenocarcinoma cell lines. Among flavan-3-ols, propelargonidin dimers were of particular interest, showing a significant correlation with cytotoxic selectivity on both gastric AGS (*R*^2^ = 0.848) and colon SW620 (*R*^2^ = 0.883) adenocarcinoma cell lines. Considering that proanthocyanidins are metabolized by the gut by the action of microbiota, these results show the evidence of the potential health effects of *U. tomentosa* proanthocyanidin extracts on gut-related diseases, such as colon cancer [15]. To further evaluate the structural-bioactivity relationship of dimeric flavan-3-ols from *U. tomentosa*, cytotoxicity tests should be carried out at equivalent proanthocyanidin concentration in the different fractions. According to these findings, further purification of valuable fractions to obtain other fractions or individual compounds would be of interest to develop novel commercial dietary ingredients or botanical drugs derived from *U. tomentosa*.

## Figures and Tables

**Table 1:** Phenolic composition of leaves fractions of *U. tomentosa.*

COMPOUNDS	LH-F3	LH-F4	LH-F5	LH-F6	LH-F7
	Concentration (µg/g Extract)				
*Hydroxybenzoic acids*					
Benzoic acid	24.7 ± 1.2	57.2 ± 3.0	401.0 ± 23	1857.1 ± 59	223.1 ± 11
Salicylic acid	9.2 ± 0.6	81.05 ± 4.0	23.7 ± 0.8	1.6 ± 0.0	nd
4-Hydroxybenzoic acid	83.9 ± 3.1	254.35 ± 9	78.1 ± 4.5	20.8 ± 1.9	5.8 ± 0.5
Protocatechuic acid	147.9 ± 6	117.5 ± 2	28.0 ± 2.1	15.9 ± 0.7	4.5 ± 0.2
Gallic acid	23.2 ± 2.4	11.5 ± 1.1	1.6 ± 0.2	1.2 ± 0.1	nd
Vanillic acid	29.2 ± 0.6	63.6 ± 1.7	63.8 ± 4.3	11.5 ± 0.8	nd
Syringic acid	2.2 ± 0.1	31.4 ± 0.8	49.2 ± 0.8	11.9 ± 0.8	nd
∑ *Hydroxybenzoic acids*	320.3	616.6	645.4	1920.0	233.4
*Hydroxycinnamic acids*					
*p*-Coumaric acid	4.9 ± 0.3	52.2 ± 2.1	178.7 ± 11	213.6 ± 8	11.0 ± 0.7
Caffeic acid	12.7 ± 0.6	32.7 ± 1.4	12.0 ± 0.9	4.6 ± 0.1	2.1 ± 0.1
Ferulic acid	nd	5.5 ± 0.1	146.0 ± 4	528.3 ± 13	49.5 ± 2.3
Isoferulic acid	nd	9.0 ± 0.1	35.2 ± 0.8	151.0 ± 3.3	46.8 ± 3.5
∑ *Hydroxycinnamic acids*	17.6	99.4	371.9	897.5	109.4
*Flavan-3-ols: monomers*					
(+)-Catechin	2285.7 ± 165.3	676.6 ± 24.4	140.3 ± 6.8	50.2 ± 0.2	9.9 ± 0.4
(−)-Epicatechin	3854.9 ± 287.1	2597.0 ± 191.8	641.9 ± 56.1	165.5 ± 4.9	63.0 ± 2.4
∑ *Monomers*	6140.6	3273.6	782.2	215.7	72.9
*Flavan-3-ols: procyanidin dimers*					
Procyanidin B1	1875.4 ± 124.7	389.2 ± 15.9	83.1 ± 3.6	37.1 ± 1.8	9.5 ± 0.3
Procyanidin B2	3327.4 ± 260.1	1137.7 ± 86.7	188.7 ± 20.7	65.9 ± 6.2	13.7 ± 1.4
Procyanidin B3	5071.0 ± 273.4	992.9 ± 26.3	175.9 ± 10.1	97.0 ± 8.5	28.2 ± 1.5
Procyanidin B4	9885.9 ± 490.1	3415.9 ± 73.9	500.7 ± 31.7	203.2 ± 17.7	54.4 ± 5.0
Procyanidin B5	370.4 ± 15.4	801.8 ± 27.6	484.1 ± 29.4	76.4 ± 6.2	nd
Procyanidin B7	760.0 ± 51.9	440.5 ± 24.8	73.5 ± 1.3	19.3 ± 0.5	nd
Procyanidin B (5.40 min)	616.6 ± 45.9	510.0 ± 36.0	91.6 ± 3.0	nd	nd
Procyanidin B (9.21 min)	49.1 ± 0.0	156.0 ± 1.5	70.8 ± 2.4	15.7 ± 1.6	nd
*∑ Procyanidin dimers*	21,955.8	7844	1668.4	514.6	105.8
*Flavan-3-ols: propelargonidin trimers*					
Propelargonidin dimer (4.36 min)	9611.4 ± 606.2	3470.3 ± 173.6	679.4 ± 31.7	273.3 ± 16.6	76.5 ± 5.1
Propelargonidin dimer (4.97 min)	5776.7 ± 381.7	4734.4 ± 227.0	1098.1 ± 81.9	279.5 ± 20.4	59.3 ± 1.2
Propelargonidin dimer (5.57 min)	4806.7 ± 284.9	6066.8 ± 218.7	2410.4 ± 189.5	382.3 ± 23.5	49.7 ± 2.6
Propelargonidin dimer (9.21 min)	111.5 ± 11.7	793.4 ± 19.6	1971.8 ± 73.1	1376.3 ± 78.1	66.0 ± 5.9
∑ *Properlargonidin dimers*	20,306.3	15,064.9	6159.7	2311.4	251.5
*Flavan-3-ols: procyanidin trimers*					
Trimer T2	291.7 ± 13.1	nd	nd	nd	nd
Procyanidin C1	799.0 ± 43.0	243.0 ± 23.5	57.5 ± 1.0	nd	nd
Trimer B (4.51 min)	1980.7 ± 58.2	327.6 ± 32.5	76.1 ± 8.2	nd	nd
∑ *Procyanidin trimers*	3071.4	570.6	133.6	nd	nd
*Flavalignans*					
Cinchonain (7.30 min)	1156.3 ± 104.2	4031.0 ± 231.7	2392.7 ± 222.5	418.8 ± 21.8	19.0 ± 0.1
Cinchonain (9.00 min)	713.2 ± 58.4	3823.0 ± 149.3	7342.0 ± 495.8	3656.1 ± 192.9	188.7 ± 3.7
Cinchonain (9.24 min)	595.9 ± 52.6	2856.4 ± 125.1	7488.5 ± 693.7	4785.4 ± 302.6	264.8 ± 5.9
Cinchonain (12.22 min)	251.2 ± 21.1	230.2 ± 12.0	2154.1 ± 165.6	8171.1 ± 417.5	1908.0 ± 7.9
*∑ Flavalignans*	2716.6	10,940.6	19,377.3	17,031.4	2380.5

nd—not detected.

**Table 2:** Total proanthocyanidins (UPLC/TQ-ESI-MS analysis) and antioxidant activity of fractions from *U. tomentosa*.

Fractions	Total Proanthocyanidins ^1^ (µg/g Extract)	ORAC Value (mmol TE/g Extract)
LH-F3	48,049.9	11.28 ± 0.06
LH-F4	34,420.1	8.28 ± 0.09
LH-F5	27,339.1	7.38 ± 0.06
LH-F6	19,857.2	7.06 ± 0.02
LH-F7	2738.0	3.37 ± 0.10

^1^ Σ = [procyanidin dimers + propelargonidin dimers + procyanidin trimers + flavalignans-cinchonains] contents (µg/g extract) (Table 1).

**Table 3:** Cytotoxicity of *U. tomentosa* fractions to gastric (AGS) and colon (SW620) adenocarcinoma cells, as well as to control Vero cells.

Sample	AGS Cells IC_50_^1^ (SI) ^2^	SW620 Cells IC_50_^1^ (SI) ^2^	Vero Cells IC_50_^1^
LH-F3	75.6 ± 4.2 (6.6)	71.4 ± 4.6 (7.0)	>500
LH-F4	128 ± 4 (3.9)	103 ± 8 (4.9)	>500
LH-F5	97.6 ± 0.9 (3.9)	86.2 ± 0.9 (4.4)	377 ± 23
LH-F6	313± 6 (0.8)	209 ± 5 (1.3)	261 ± 29
LH-F7	358 ± 24 (1.1)	232 ± 1 (1.7)	400 ± 31

^1^ µg/mL; ^2^ Selectivity Index.

## Supplementary Materials

The following are available online at www.mdpi.com/2076-3921/6/3/60/s1.
