# Supplementary material for: Fractioning of Proanthocyanidins of Uncaria tomentosa. Composition and Structure-Bioactivity Relationship
Source: Antioxidants (Basel). 2017 Jul 28;6(3):60. doi: 10.3390/antiox6030060 (PMC5618088; doi:10.3390/antiox6030060)
Supplement: Supplementary file 1 [file antioxidants-06-00060-s001.pdf]

Supplementary Material

Table S1. MS/MS parameters for the identified polyphenols.

Compound	MRM Transition (*m*/*z*)	Collision Energy (V)	Cone Voltage (V)
Benzoic acid	121 > 77	12	27
Salicylic acid	137 > 93	17	27
4-Hydroxybenzoic acid	137 > 93	12	27
Protocatechuic acid	153 > 109	12	30
Gallic acid	169 > 125	15	33
Vanillic acid	167 > 152	12	30
Syringic acid	197 > 182	12	30
p-Coumaric acid	163 > 119	14	30
Caffeic acid	179 > 135	16	35
Ferulic acid	193 > 134	16	30
Isoferulic acid	193 > 134	14	27
(+)-Catechin	289 > 245	16	40
(−)-Epicatechin	289 > 245	14	38
Procyanidin dimers	577 > 289	25	45
Procyanidin trimers	865 > 577	25	50
Propelargonidin dimers	561 > 289	25	45
Flavalignans-cinchonains	451 > 341	25	45

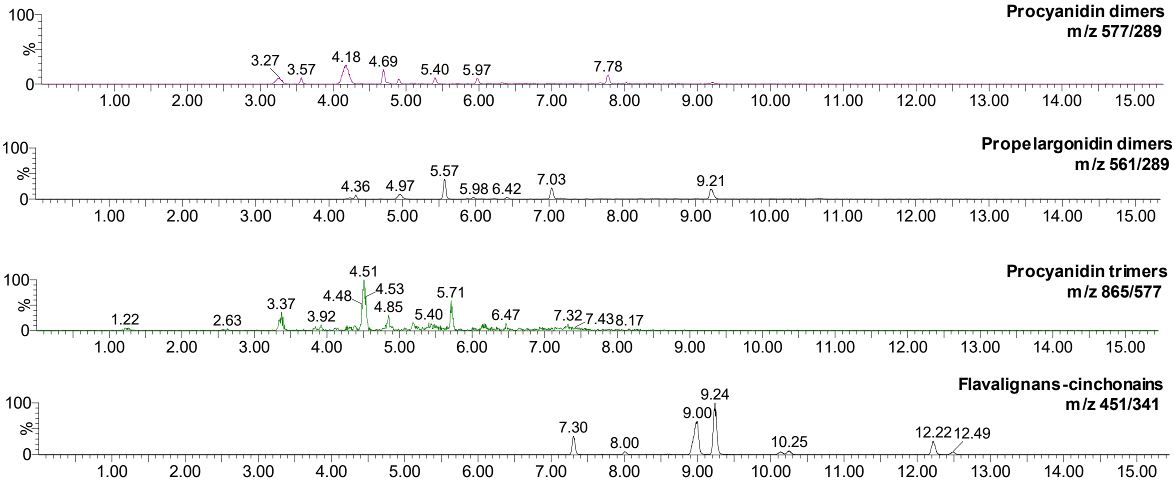

Figure S1. MRM chromatograms (UPLC-DAD-ESI-TQ-MS) for proanthocyanidins in *U. tomentosa* leaves fractions.

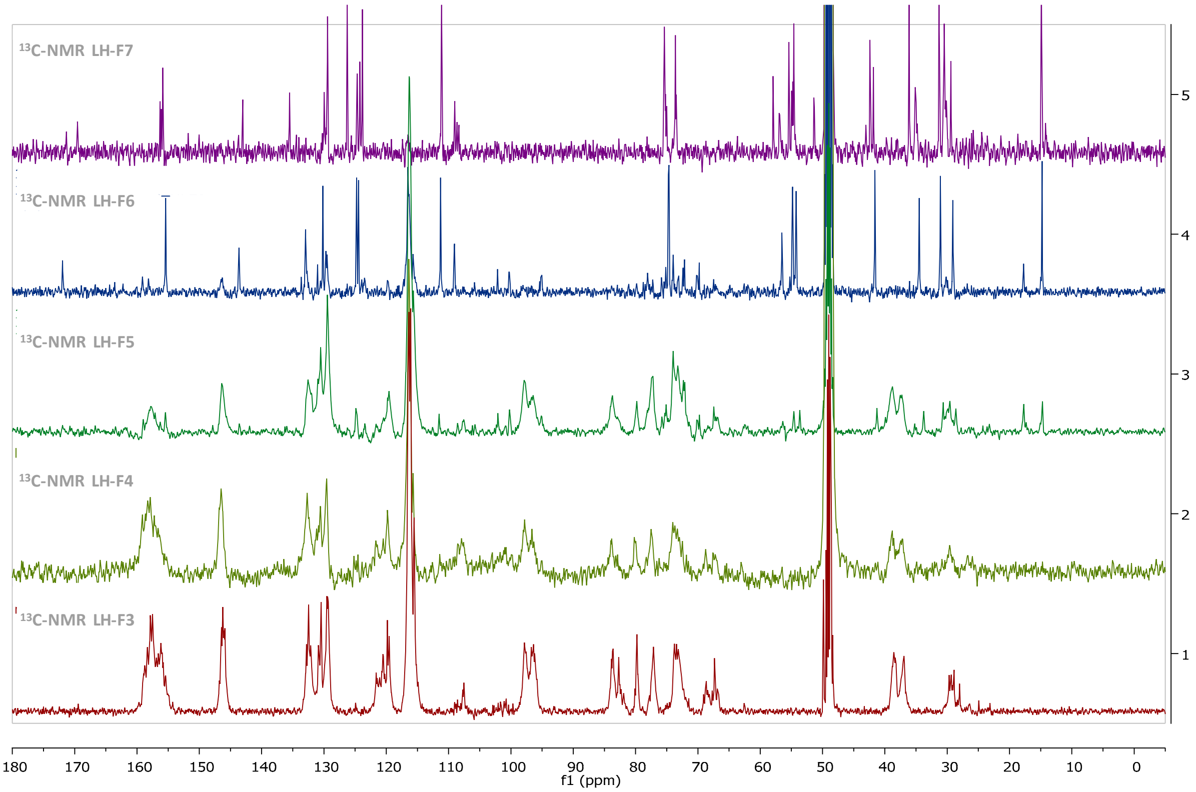

Figure S2. ^{13}C-NMR (MeOD) for polyphenolic fractions LH-F3 a LH-F7 from *U. tomentosa* leaves

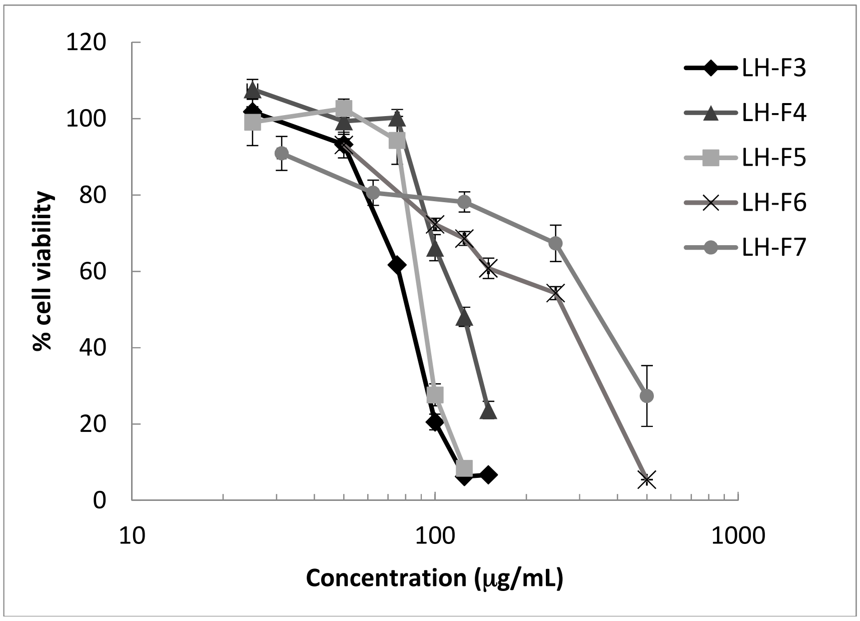

Figure S3. Dose-response curves of cytotoxicity of *U. tomentosa* fractions on AGS gastric adenocarcinoma[1].

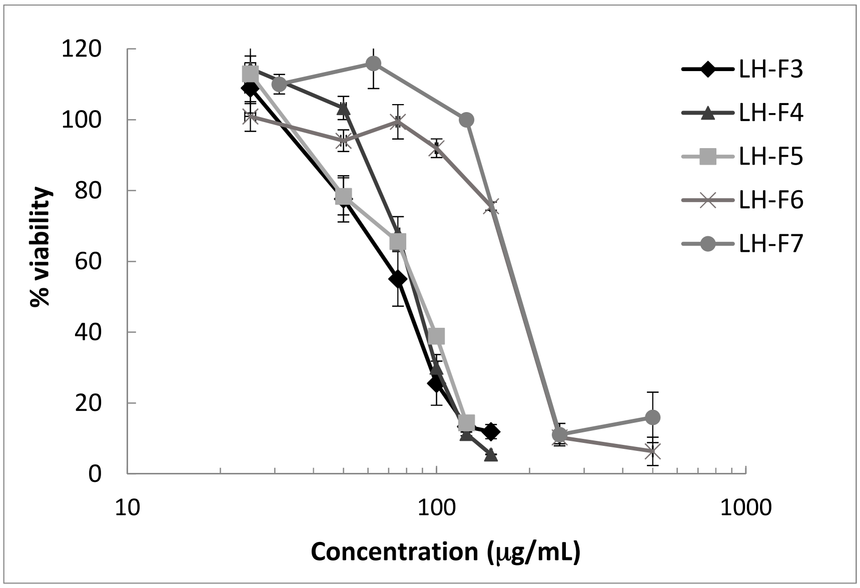

Figure S4. Dose-response curves of cytotoxicity of *U. tomentosa* fractions on SW620 colon adenocarcinoma[1].
[1] Results represent the mean ± SE of triplicates of one representative experiment of each fraction.
